# Calls for Stricter Legislation and Fear in the European Immigrant Community: Reflections of the Public Charge Debate Ongoing in the United States: Comment on "A Crisis of Humanitarianism: Refugees at the Gates of Europe"

**DOI:** 10.15171/ijhpm.2019.97

**Published:** 2019-11-02

**Authors:** Jimmy Efird

**Affiliations:** Cooperative Studies Program Epidemiology Center, Health Services Research and Development, DVAHCS (Duke Affiliate), Durham, NC, USA.

**Keywords:** Refugees, Immigrants, Public Charge

## Abstract

In the editorial, "A Crisis of Humanitarianism: Refugees at the Gates of Europe," Marianna Fotaki elegantly highlights the changing dynamics of governmental policy toward refugees, forced migrants into Europe and the move away from the principles of humanitarianism.^1^ The perceived threats to economy, security, and concerns of globalization and multiculturalism often are manifested as a "cry of wolf " about alleged health risks. This in effect has raised concerns of inadmissibility on health-related grounds and calls for stricter legislation for determining who is eligible for legal permanent residence, precipitated in part by the "public charge" debate occurring in the United States.^2^ As Marianna notes "anti-migration rhetoric is now a permanent fixture of European politics."

While the definition of public charge varies across countries, the term usually refers to immigrants who predominately depend upon their host governments (owing to advanced age, poor health, low income, large family size, and a lack of formal education or applied skills) for subsistence.^3^ Those who are deemed to be self-sufficient are not considered as a public charge. In most cases, charge is in the form of public cash assistance or long-term institutional care. However, as governments face dwindling cash reserves with the influx of individuals from war zones, natural disasters, drug violence, sex trafficking, and genocide, policy-makers are increasingly reconsidering “non-cash” programs to be on the table when negotiating legislation.

Non-cash programs are designed to aid vulnerable immigrant groups to become self-sufficient by improving health, nutrition, and independence of welfare payments.^4^ They include public housing; supplemental food assistance for infants, children, and mothers; children health insurance; education; and job training. Although refugees and asylum applicants typically are exempt from public charge rules, the rigor of legally proving one’s status often is an insurmountable barrier. Changes to public charge legislation, especially when poorly conceived and not carefully thought through over the long run, pose risk of discretionary decision-making on the part of immigration officers. Assessing the “totality of circumstances” already is a complex and subjective process, with adjudicating officers given tremendous lead way in determining who will be designated as a public charge.

Fear of legal exposure, deportation, or delays in obtaining legal permanent residence, even when not actually merited by public charge legislation, may result in a reluctance of immigrants to seek needed medical attention and to forego enrollment in preventive public health programs. This may lead to increased disease outbreaks, with financial consequences to the greater community that dwarf the costs of a carefully orchestrated plan for the health and well-being of immigrants. Historically, this was the case for the San Francisco smallpox plague of 1900-1904, where families secretly buried their dead, out of fear of economic discrimination and public reprisal.^5^ The inadequate vaccination for measles represents another legitimate public health example in immigrant communities.

Europeans will benefit by carefully following and learning from the ongoing public charge debate occurring in the United States, hopefully avoiding many of the unsubstantiated claims and media distortions regarding public health risk. A proactive (vs. reactionary) approach by governments when adopting such policies will facilitate a better understanding of the long-term or unintended consequences of any proposed legislative changes. For example, policy-makers may inadvertently create an underclass of individuals who will be unable to fully participate or contribute to their host country, exacerbating the long-term impacts including costs and public health. These policies also can change which immigrants are welcomed (eg, wealthy and educated individuals), therefore increasing inequities within countries.

Fotaki is correct when stating that the issues underlying refugee and immigrant needs “concerns all of us” and requires collective political action. Simply ignoring the health and welfare needs of this at-risk population is counter productive in a modern civilized society. As she concludes, “in offering such protection, we recognize our dependence on others for our own survival as individuals and social beings.”

## Ethical issues

Not applicable.

## Competing interests

Author declares that he has no competing interests.

## Author’s contribution

JE is the single author of the paper.

## Disclaimer

The views expressed in this commentary do not necessarily reflect the position or policy of CSPEC/DVAHCS, the US Department of Defense, or the US Government.
